# Identification of LARS as an essential gene for osteosarcoma proliferation through large-Scale CRISPR-Cas9 screening database and experimental verification

**DOI:** 10.1186/s12967-022-03571-9

**Published:** 2022-08-12

**Authors:** Wenhao Chen, Yuxiang Lin, Meichen Jiang, Qingshui Wang, Qiang Shu

**Affiliations:** 1 Department of Orthopedics, The Children’s Hospital, Zhejiang University School of Medicine, National Children’s Regional Medical Center, National Clinical Research Center for Child Health, 3333 Bingsheng Road, Hangzhou, 310052 Zhejiang Province China; 2 Department of Breast Surgery, Fujian Medical University Union Hospital, 29 Xinquan Road, Fuzhou, 350001 Fujian Province China; 3 Department of General Surgery, Fujian Medical University Union Hospital, Fuzhou, 350001 Fujian Province China; 4 Breast Cancer Institute, Fujian Medical University, Fuzhou, Fujian Province China; 5 Department of Pathology, Fujian Medical University Union Hospital, 29 Xinquan Road, Fuzhou, 350001 Fujian Province China; 6 College of Life Sciences, Fujian Normal University, 8 South Xuefu Road, Fuzhou, 350117 Fujian Province China; 7 The Children’s Hospital, Zhejiang University School of Medicine, National Children’s Regional Medical Center, National Clinical Research Center for Child Health, 3333 Bingsheng Road, Hangzhou, 310052 Zhejiang Province China

**Keywords:** Osteosarcoma, Depmap, CRISPR-Cas9, LARS, Proliferation

## Abstract

**Background:**

Osteosarcoma is one of the most malignant tumors, and it occurs mostly in children and adolescents. Currently, surgery and chemotherapy are the main treatments. The recurrence rate is high and the prognosis is often poor. Finding an effective target gene therapy for osteosarcoma may effectively improve its prognosis.

**Method:**

In this study, genes essential for the survival of osteosarcoma cells were identified by genome-wide screening of CRISPR-Cas9 based on the DepMap database. The expression of these essential genes in osteosarcoma patients’ tissues and normal tissues was identified in the GSE19276 database. Functional pathway enrichment analysis, protein interaction network construction, and LASSO were performed to construct a prognostic risk model based on these essential genes. CCK8 assay was used to detect the effect of essential gene-LARS (Leucyl-TRNA Synthetase 1) on the proliferation of osteosarcoma.

**Results:**

In this study, 785 genes critical for osteosarcoma cell proliferation were identified from the DepMap. Among these 785 essential genes, 59 DEGs were identified in osteosarcoma tissues. In the functional enrichment analysis, these 59 essential genes were mainly enriched in cell cycle-related signaling pathways. Furthermore, we established a risk score module, including LARS and DNAJC17, screened from these 59 genes, and this module could divide osteosarcoma patients into the low-risk and high-risk groups. In addition, knockdown of LARS expression inhibited the proliferative ability of osteosarcoma cells. A significant correlation was found between LARS expression and Monocytic lineage, T cells, and Fibroblasts.

**Conclusion:**

In conclusion, LARS was identified as an essential gene for survival in osteosarcoma based on the DepMap database. Knockdown of LARS expression significantly inhibited the proliferation of osteosarcoma cells, suggesting that it is involved in the formation and development of osteosarcoma. The results are useful as a foundation for further studies to elucidate a potential osteosarcoma diagnostic index and therapeutic targets.

**Supplementary Information:**

The online version contains supplementary material available at 10.1186/s12967-022-03571-9.

## Introduction

Osteosarcoma is the predominant type of malignant bone tumor, affecting mostly children and adolescents [1]. The advancement of surgical resection with adequate surgical margins and neoadjuvant chemotherapy has increased the survival rate from less than 20% in 1970s to 60–70% nowadays [2]. However, clinically detectable metastases are found at diagnosis among approximately 15–20% of patients with osteosarcoma [3]. The most common metastatic disease occurs in lungs, whereas the second most common site of metastasis is bone. The survival of patients with unresectable, metastatic or relapsed osteosarcoma are still unsatisfactory, with an overall 5-year survival rate of less than 20% [4]. Current standard treatment seems to be ineffective against these recurrent or metastatic osteosarcomas [5]. Therefore, there is an urgent demand in identification of therapeutic targets for advanced osteosarcoma patients.

Recently, high-throughput screening projects such as DepMap based on RNA interference silencing and CRISPR-Cas9 knockout techniques have emerged to be a powerful approach to identify potential dependency genes that are crucial to tumor survival, metastasis or recurrence [6–8]. These identified tumor vulnerabilities could be potential therapeutic targets. CERES algorithm was developed to calculate gene-knockout effects. CERES scores represent the median effects of vital genes and nonessential genes for per cell line [9]. Genes that are only essential in a few cell lines are recognized as better drug targets because it is unlikely to cause toxicity in normal tissues after inhibiting their function. Furthermore, investigation and validation of prognosis-predictive value of these cancer essential genes may allow better clinical decision making for orthopedic surgeons.

In this study, we aimed to identify candidate essential genes that are essential to the survival of osteosarcoma, by screening DepMap database, functional enrichment and LASSO analyses. Then, a predictive model based on these essential genes for overall survival was constructed. Finally, cell proliferation was further evaluated in response to knocking down the candidate gene to determine its oncogenic potential.

## Methods

### Identification of genes essential for survival of osteosarcoma cells through DepMap database

To identify potential cancer therapeutic targets, the Broad Institute constructed the DepMap (Cancer Dependency Map) database, which contains gene dependency information of more than 700 human tumor cell lines of different tissue origins and gene expression, gene copy number and gene mutation information of more than 1,000 tumor cells (https://depmap.org/portal/). Through the DepMap database, we can know the gene dependence of different cell lines. Genes with amplified gene copy number can cause severe DNA damage during CRISPR-Cas9 cleavage and cause cell growth arrest or apoptosis, which can lead to false positives. Therefore, in the process of using CRISPR-Cas9 technology to evaluate the dependence of cells on genes, the DepMap database comprehensively considered the two factors of gene copy number and sgRNA loss, and set a new parameter CERES as a parameter to measure the degree of gene necessity. The principle of CERES is that the fewer cells that carry the gene's sgRNA survive, the fewer copies of the gene are in the cell, and the more the cells are dependent on the gene. A negative CERES score indicates that knocking out the gene inhibits the survival and proliferation of the cell lines. In the study, we defined genes as an essential gene with CERES scores < − 1 in more than 75% of osteosarcoma cell lines [10].

### Identification of differentially expressed genes (DEGs)

Limma is a differential expression screening method based on generalized linear models. In this study, the R software package limma (version 3.40.6) was used for differential analysis to obtain the differential genes between the osteosarcoma group and the normal control group. Genes with a fold-change (FC) > 1.5 or < 0.67 (corresponds to |log2FC| ≥ 0.585) and *p*-value < 0.05 were considered significantly DEGs.

### Clinical data collection and extraction

MRNA expression profiles of GSE19276, GSE21257 and GSE39055 were downloaded from the GEO database (http://www.ncbi.nlm.nih.gov/geo/). The dataset for GSE19276 includes 44 osteosarcoma samples and 5 normal tissue samples. GSE21257 and GSE39055 incorporate 53 patients and 37 patients with follow-up information, respectively.

### Patients and specimens

A total of 18 osteosarcoma specimens and 18 paired normal specimens from Fujian Medical University Union Hospital between August 2019 and August 2021 were collected. The project was approved by the Research Ethics Committee of Fujian Medical University Union Hospital. All patients provided written informed consent under an institutionally approved protocol.

### GSEA analysis

We obtained the GSEA software (version 3.0) from the GSEA (http://software.broadinstitute.org/gsea/index.jsp) website, and divided the samples into high expression groups (50%) and low expression groups according to the expression level of LARS group (50%) to assess relevant pathways and molecular mechanisms. *P* value of < 0.05 was considered statistically significant.

### ROC analysis

We performed ROC analysis using the R software package pROC (version 1.17.0.1) to obtain the AUC. Specifically, we obtained the patient's follow-up time and risk score, and used the roc function of pROC to perform the ROC analysis of 1-year, 3-year, and 5-year.

### Gene enrichment analysis

The DAVID (Database for Annotation, Visualization and Integrated Discovery) database can provide biological meaning for genes (https://david.abcc.Ncifcrf.gov/).In the research, KEGG-pathway and GO-BP enrichment analysis of identified DEGs was performed using the DAVID database.

### Protein–protein interaction (PPI) network analysis of DEGs

PPI analysis of DEGs was analyzed using Metascape and the node score was calculated and obtained in Metascape (http://metascape.org). MCODE (Molecular Complex Detection) algorithm was performed to identify the densely connected network neighborhoods. Each MCODE component is marked with a different color and characterizes their biological significance.

### Least absolute shrinkage and selection operator (LASSO) analysis

In this study, we used the R software package glmnet to integrate survival time, survival status, and gene expression data for regression analysis using the lasso-cox method.

### Drug sensitivity evaluation

GSCALite is a website used for the analysi of drug sensitivity and genomic cancer (http://bioinfo.life.hust.edu.cn/web/GSCALite/). In the research, GSCALite was used to evaluate the drug sensitivity of LARS and DNAJC17 to identify potential compounds for treatment.

### Cells culture and transfection

The cell lines Saos-2 and U-2 OS were obtained from ATCC (American Type Culture Collection). Saos-2 and U-2 OS cells were cultured in DMEM medium (Gibco) containing 10% fetal bovine serum (FBS, BI, Kibbutz Beit Haemek, Israel), 100 U/mL penicillin and 0.1 mg/mL streptomycin (BBI life sciences, shanghai, China) andat 37 °C in a humidified incubator with 5% CO_2_. The sequence of shRNAs targeting LARS was cloned into pLVX vector. The sequence of LARS shRNA1 was 5′-CTGGACATCACTTGTTTCT-3′; The sequence of LARS shRNA2 was 5′-AAATGAAGGCGTCCATTCA-3′. The transfection was performed using lipofectamine 2000 (Invitrogen, Carlsbad, CA, USA) according to the manufacturer’ s guidelines.

### RNA isolation and RT-qPCR

Total RNA was extracted from Saos-2 and U-2 OS cells using TRIzol (Invitrogen, CA, USA), and was reverse transcripted with mRNA reverse transcription kit (Takara, Japan). Specific primers for RT-qPCR were performed to detect the mRNA expression of LARS. All primers were synthesized by Sangon Biotech (Shanghai, China). LARS-forward primer 5′-ATGGCGGAAAGAAAAGGAACAG-3′ and LARS-reverse primer 5′-CAGGCCAAAGGGAAACAGACAAC-3′. GAPDH primers were as follows; GAPDH-forward primer 5′-GCGGGGCTCTCCAGAACATCAT-3′ and GAPDH-reverse primer 5′-CCAGCCCCAGCGTCAAAGGTG-3′. Relative quantification was performed by the 2-^ΔΔCt^ method.

### Western blot

Cells were lysed in RIPA lysis buffer (Roche Ltd, Basel, Switzerland) containing protease inhibitors. Protein concentration in the lysates was measured by Micro BCA Protein Assay Kit (Pierce Biotechnology, IL, US). Samples were separated on 10% SDS-PAGE and subsequently transferred to Amersham Protran nitrocellulose membranes (GE Healthcare Life Sciences, Fairfield, USA). The nitrocellulose membranes were then incubated with primary antibodies for the target proteins LARS and GAPDH (Proteintech, Wuhan, China) at a dilution of 1:1000 and 1:5000 for 2 h, respectively. The proteins were detected and quantified using the Odyssey^®^ CLx Infrared Imaging System (LI-COR Biosciences, NE, USA).

### Immunohistochemistry (IHC) staining analysis

We conducted IHC staining analysis to measure the protein expression of LARS in OS tissues and adjacent normal tissues according to the standard immunoperoxidase staining procedure. Slides were incubated with anti-LARS (21146-1-AP, Proteintech, Wuhan, China, diluted 1:400). The IHC staining scores of LARS were evaluated by two independent pathologists. The percentage of stained positive cells was calculated from 1 to 4: 1, 0–25%; 2, 26–50%; 3, 51–75%; and 4, 75–100%. The staining intensity score was ranged from 0 to 3: 0, no staining; 1, weak staining; 2, moderate staining; and 3, strong staining. The percentage of positive tumor cells and the staining intensity were multiplied to produce a weighted score for each case.

### CCK-8 assay

Saos-2 and U-2 OS cells were seeded into 96-well plates (2 × 10^4^ cells/well) and cultured for 24 h, 48 h, and 72 h. Four hours before absorbance measuring, 10 μL CCK-8 solution was added. The absorbance was measured at 450 nm with a microplate reader after incubating at 37 °C for 2 h.

### Statistical analysis

In the study, a statistical correlation was calculated by t-test. Overall survival (OS) was evaluated by the Kaplan–Meier method, and survival curves were compared by log-rank test. Two-way ANOVA was used to analyze CCK8 assay. All *p* values < 0.05 were considered statistically significant.

## Results

### Identifying candidate genes that are critical for the survival of osteosarcoma cells

Project Achilles knocks out each gene individually by using a genome-scale CRISPR-Cas9 tool and identifying candidate genes that are critical for the survival of tumors. Taking advantage of RNA-Seq data and Project Achilles from osteosarcoma patients, we can pinpoint essential genes responsible for osteosarcoma malignancy. Candidate genes were defined as essential genes responsible for osteosarcoma malignancy with a CERES score of <  − 1 across 75% of osteosarcoma cell lines (n = 8), including Saos-2, G-292, U-2 OS, SJSA-1, 143B, HuO9, OS252, and HS-Os-1. In total, 785 genes were finally found to be crucial for maintaining survival in the 8 osteosarcoma cell lines (Additional file 1: S1).

**Fig. 1:**
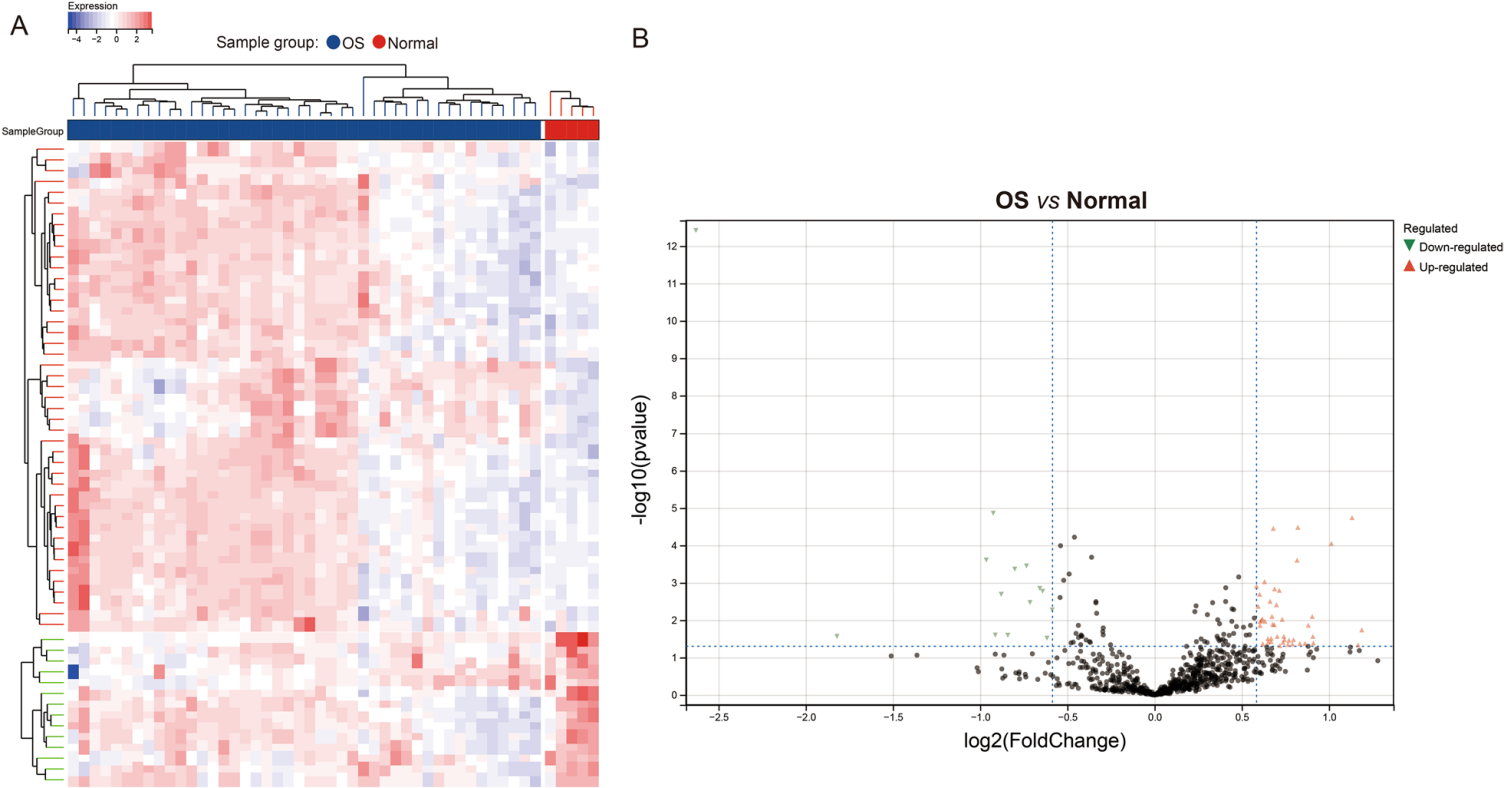
Differential expression analysis of the 785 essential genes in osteosarcoma tissues. **A** Heatmap and **B** volcano plot showing differential expression of 785 essential genes in osteosarcoma tissue (n = 44) and normal tissues (n = 5) on GSE19276 database

To identify which candidate genes among these 785 genes were dysregulated expression in osteosarcoma tissues, DEG (Differentially Expressed Genes) analyses were performed to compare osteosarcoma tissues (n = 44) with normal tissues (n = 5) in the GSE19276 database. Fifty-nine of 785 essential genes were significantly changed in osteosarcoma tissues (Fig. 1, Additional file 2: S2). Among these 59 genes, 14 genes were significantly down-regulated, and 45 genes were significantly up-regulated in osteosarcoma tissues.

**Fig. 2:**
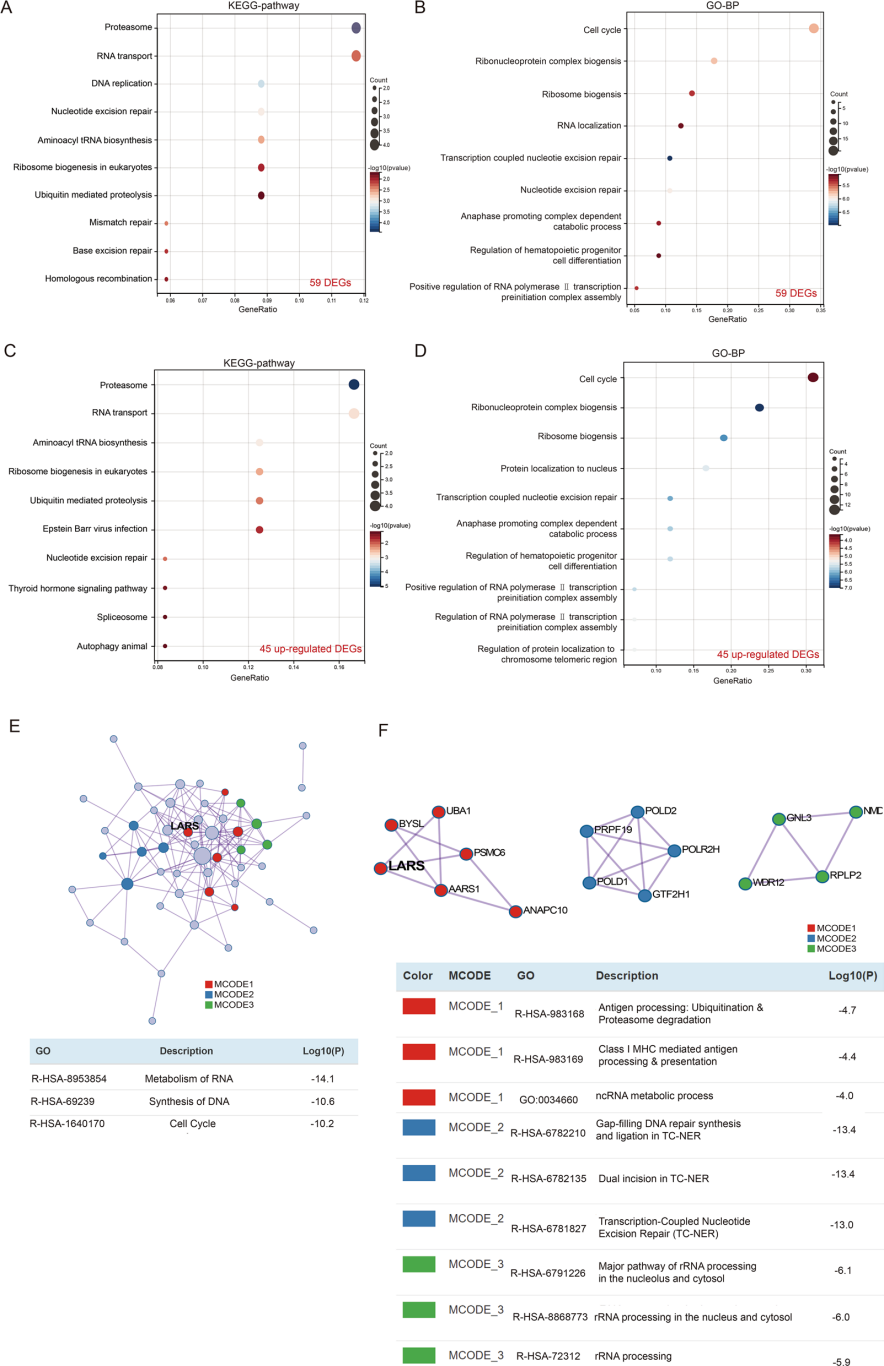
Gene enrichment analysis of the 59 differentially expressed genes. **A** KEGG pathways and **B** GO-BP terms for the 59 differentially expressed genes. **C** KEGG pathways and **D** GO-BP terms for the 45 upregulated genes. **E** Protein–protein interaction of the 59 differentially expressed genes. **F** MCODE network clustering analysis of the 59 differentially expressed genes

To identify the biological roles of these essential genes in osteosarcoma, the enrichment with KEGG-pathway and GO-BP (biological process) was analyzed. From the results of KEGG-pathway enrichment, we found that these 59 genes are mainly enriched in the proteasome, RNA transport, DNA replication, Nucleotide excision repair, aminoacyl tRNA biosynthesis, ribosome biogenesis in eukaryotes, ubiquitin mediated proteolysis, mismatch repair, base excision repair and homologous recombination (Fig. 2A). In addition, in the enrichment results of GO-BP, these genes were mainly related to the cell cycle, ribonucleoprotein complex biogenesis, ribosome biogenesis, RNA localization, transcription coupled nucleotide excision repair, nucleotide excision repair, anaphase promoting complex depend catabolic process, regulation of hematopoietic progenitor cell differentiation, and positive regulation of RNA polymerase II transcripition preinitiation complex assembly (Fig. 2B). In additional, enrichment analysis was conducted for the 45 upregulated genes and the 14 downregulated genes, respectively. However, the number of down-regulated genes is too small to perform a reliable KEGG and GO analysis. The KEGG and GO analysis were performed on only upregulated genes. From the results of KEGG-pathway enrichment, we found that these 45 upregulated genes are also mainly enriched in the proteasome, RNA transport, etc., (Fig. 2C). In the enrichment results of GO-BP, these 45 upregulated genes were also mainly related to the cell cycle, ribonucleoprotein complex biogenesis, etc. (Fig. 2D). The results showed that these essential genes were mainly associated with the cell cycle-related signaling pathway in the osteosarcoma tissues.

Next, a protein–protein interaction (PPI) network of these 59 essential genes was generated using Metascape (Fig. 2E). After the analysis of molecular complex detection, three MCODE components were obtained from the PPI network (Fig. 2F). In addition, the enrichment analysis of every MCODE component is depicted in Fig. 2D, in which the top three enrichment results are displayed. Among the three MCODE components, the most significant was MCODE1, which included six genes: LARS (Leucyl-TRNA Synthetase 1), BYSL (Bystin Like), UBA1 (Ubiquitin Like Modifier Activating Enzyme 1), PSMC6 (Proteasome 26S Subunit, ATPase 6), AARS1 (Alanyl-TRNA Synthetase 1) and ANAPC10 (Anaphase Promoting Complex Subunit 10).

### Construction of essential genes-based prognostic predictors

**Fig. 3:**
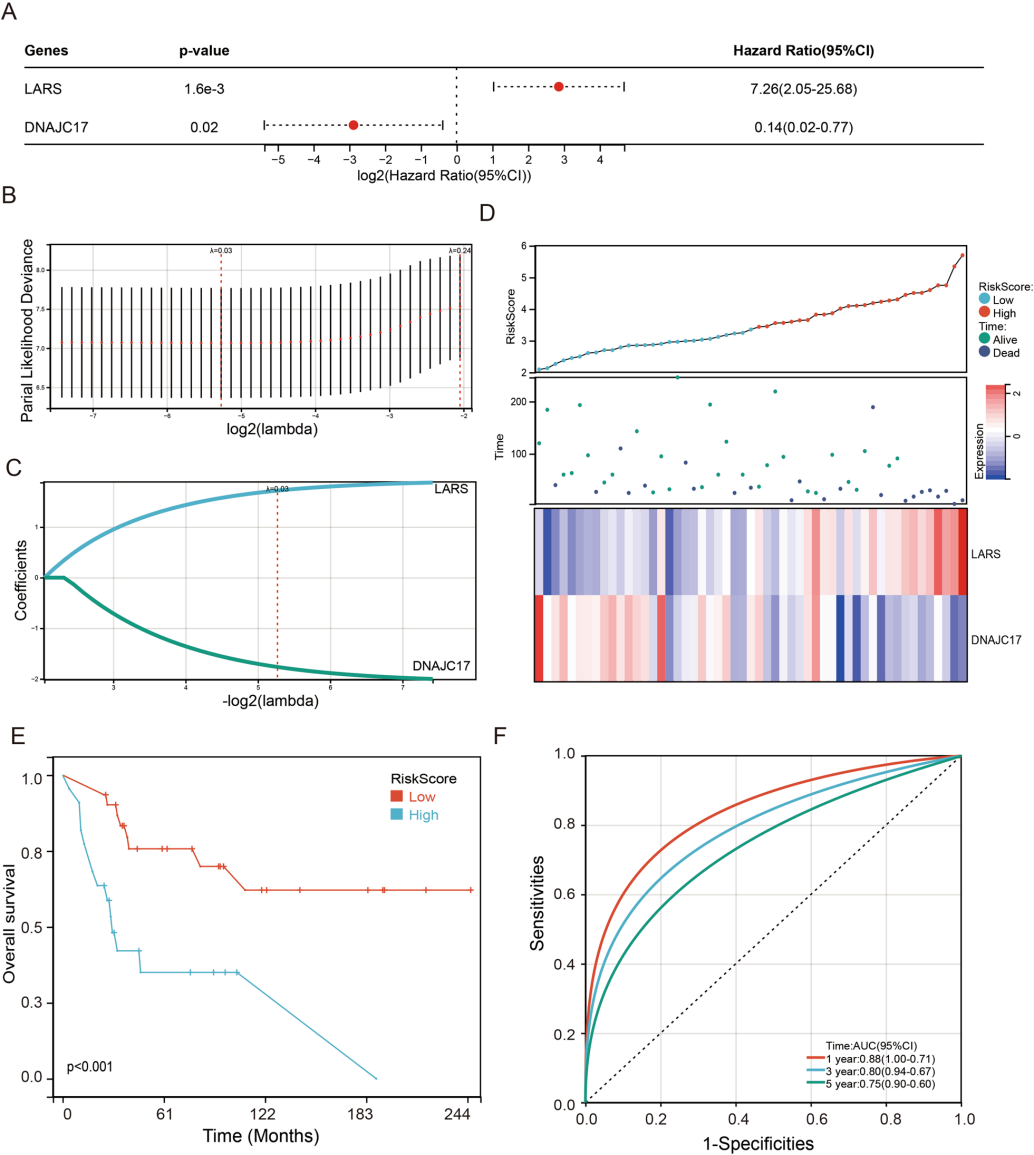
AConstruction of a risk model in osteosarcoma. **A** univariate Cox survival analysis on the 59 differentially expressed genes. **B**, **C** The LASSO-Cox regression model calculated the coefficient and achieved the risk score. **D** The distribution of risk score, survival status, and the corresponding heatmap of the gene expression levels of each patient was analyzed and plotted. **E** The prognostic value of the risk score on OS for each osteosarcoma patient by Kaplan–Meier analysis. **F** Time-dependent ROC curves for survival prediction

The prognosis of these 59 essential genes for osteosarcoma patients was further analyzed by using the univariate Cox regression analysis based on GSE21257 dataset. The results revealed that high expression of LARS was significantly negatively correlated with the prognosis of osteosarcoma patients, and low expression of DNAJC17 (DnaJ Heat Shock Protein Family (Hsp40) Member C17) was significantly negatively correlated with the prognosis of osteosarcoma patients, Next, we used the LASSO analysis of the tenfold cross-validation to analyze LARS and DNAJC17 genes (Fig. 3B), and constructed the risk score model that included LARS and DNAJC17 genes (Fig. 3C). The risk score was calculated as follows: Risk score = (LARS × 1.7203 − DNAJC17 × 1.7652). Each osteosarcoma patient was divided into low-risk and high-risk groups according to the risk score. The distribution of risk scores, survival status, and the mRNA expression of two genes in osteosarcoma patients were shown in Fig. 3D. Kaplan–Meier survival revealed that the low-risk group was correlated with a better prognosis for osteosarcoma patients (Fig. 3E). The result of the ROC analysis demonstrated that the risk score had a powerful ability to predict prognosis in osteosarcoma patients (1-year (AUC = 0.88), 3-year (AUC = 0.80), 5-year (AUC = 0.75); Fig. 3F).

**Fig. 4:**
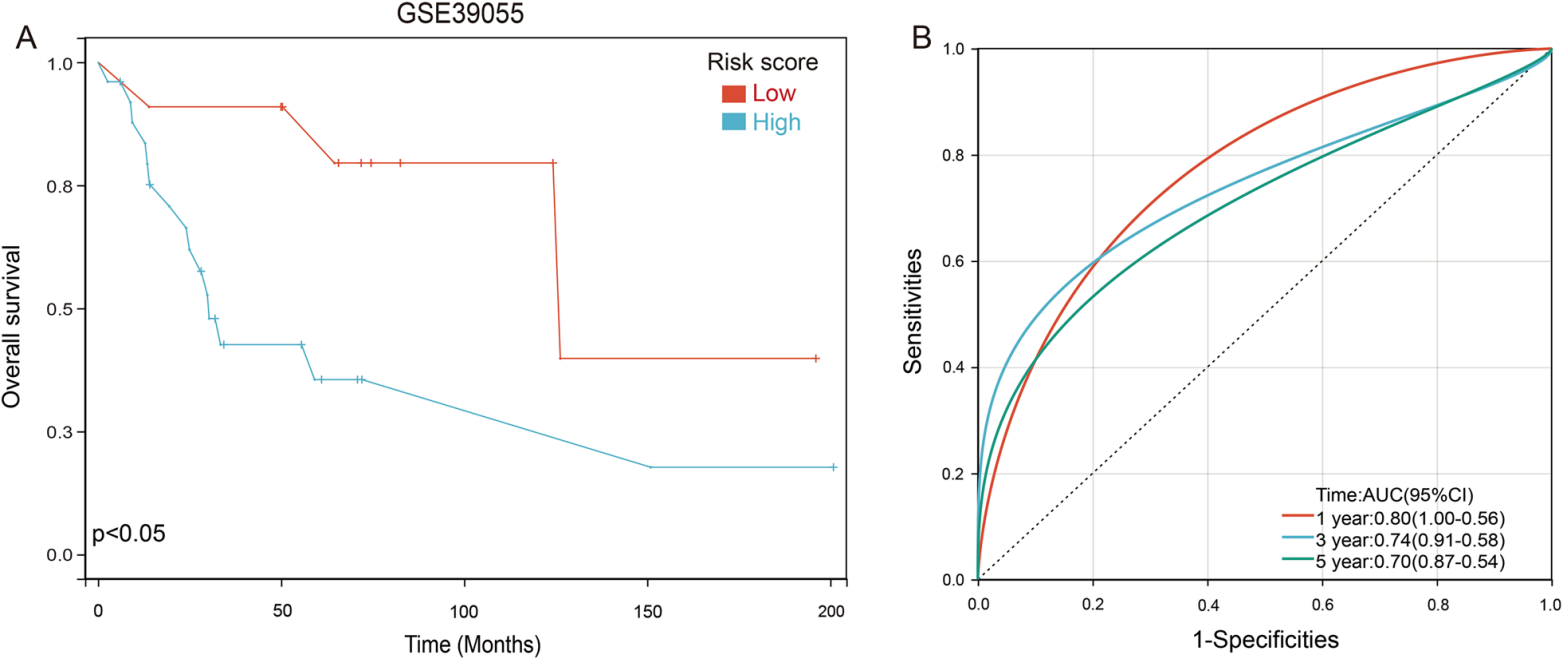
Validation of the prognosis risk model. **A** Kaplan–Meier analysis of the prognostic model by using the GSE39055 database. **B** Plotting ROC curves to assess the accuracy of the prognostic model based on GSE39055 database

To further verify the prognostic model, we downloaded 37 osteosarcoma samples with complete clinical information from GSE39055 database. Each patient was brought into the previous prognostic model to calculate the risk score. Patients were divided into high-risk and low-risk groups. Kaplan–Meier curve analysis showed that osteosarcoma patients with low-risk scores had a better overall survival than those in the high-risk-score group (Fig. 4A). The result of the ROC analysis demonstrated that the risk score had a powerful ability to predict prognosis in osteosarcoma patients (1-year (AUC = 0.80), 3-year (AUC = 0.74), 5-year (AUC = 0.70); Fig. 4B).

**Fig. 5:**
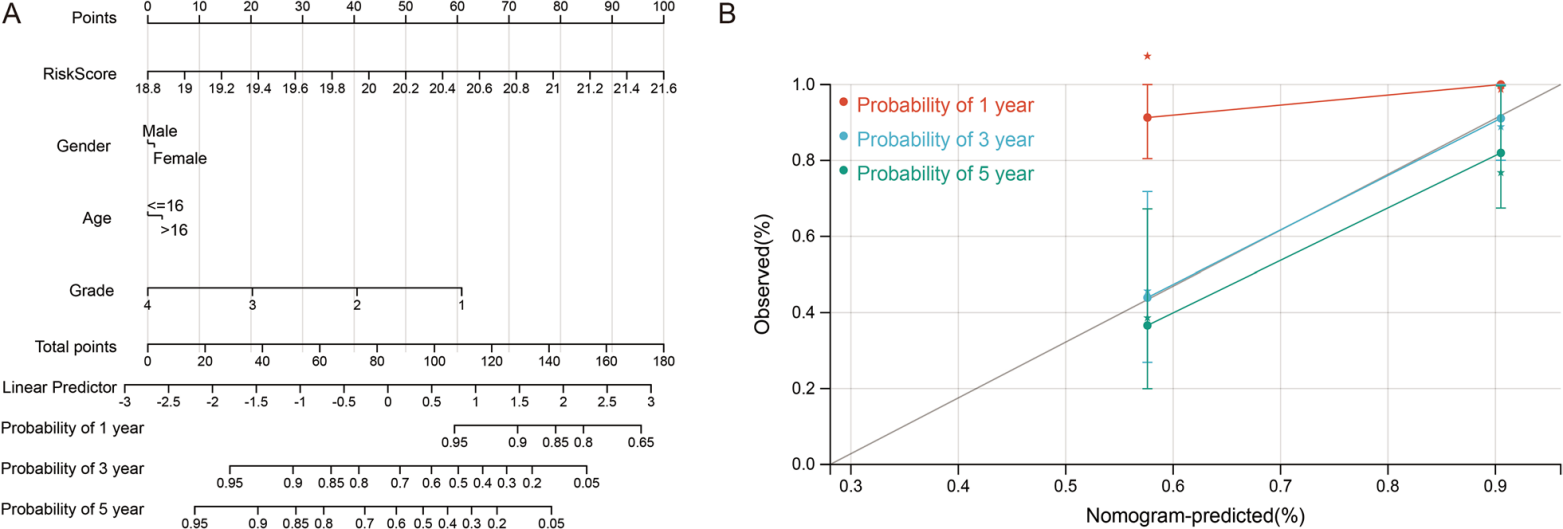
Construction of clinical prognostic prediction model. **A** The survival nomogram was set up to exactly figure up the 1-year, 3-year, and 5-year survival rate based on five variables containing gender, age, tumor grade, and risk score. **B** The comparison between the standard curve and the ideal model

Next, a nomogram model-independent predictors of OS, including risk score, gender, age, and tumor grade, was developed. The nomogram model provides a visual statistical predictive tool for the survival of osteosarcoma patients, allowing the risk-scoring model to be applied to clinical practice (Fig. 5A). The C‐index for the nomogram is 0.75, indicating the nomograph we built is reliable and accurate (Fig. 5B).

### Analysis of drug sensitivity associated with LARS and DNAJC17

**Fig. 6:**
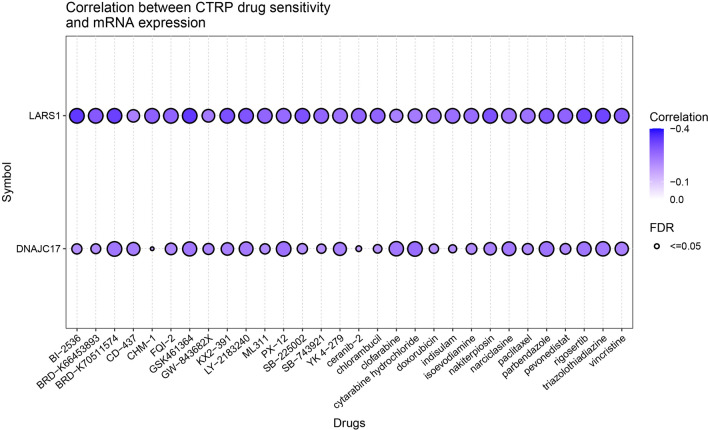
Drug sensitivity of LARS and DNAJC17 genes obtained from GSCA. The correlations between the LARS and DNAJC17 expression and drugs. Bubble size is positively correlate with the FDR significance. Black outline border indicates FDR ≤ 0.05

The GSCA database was exploited to analyze the relationship between the drug sensitivity and the expression of LARS and DNAJC17 based on the data from the Cancer Drug Sensitivity Genomics Database (GDSC) (Fig. 6, Additional file 1: S3). The result revealed that high LARS expression was associated with higher drug sensitivity to BRD-K30748066, Tozasertib, BI-2536, GSK461364, BRD-K70511574, Triazolothiadiazine, Rigosertib, FQI-1, KX2-391 and SB-225002 (R < − 0.3, p < 0.05). There was no significant correlation between the expression of DNAJC17 and the drug sensitivity.

### Knockdown of LARS inhibits osteosarcoma cell proliferation

**Fig. 7:**
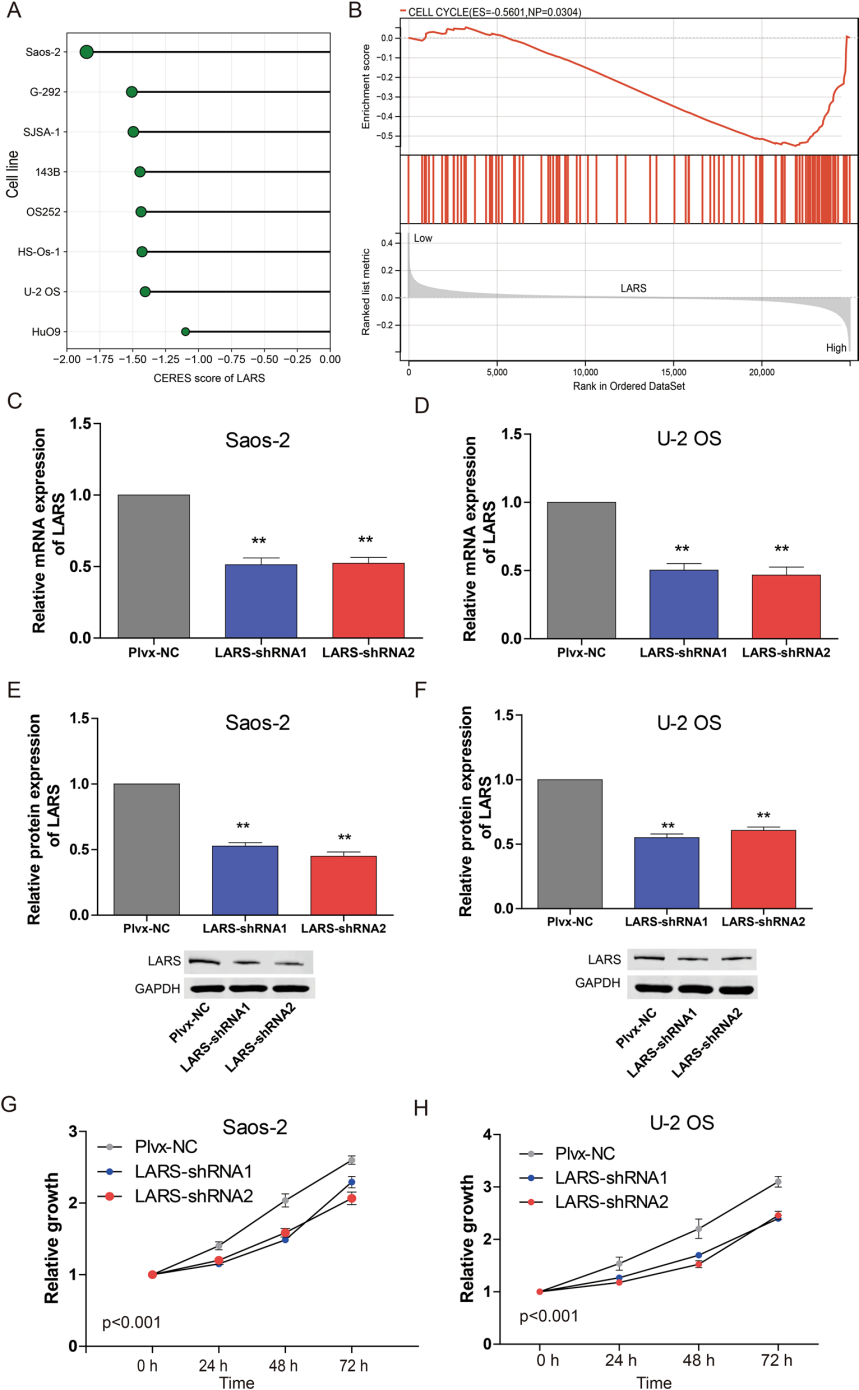
The function of LARS in osteosarcoma cells. **A** The CERES score of LARS in 8 osteosarcoma cell lines. **B** GSEA analysis suggested that the high expression of LARS is related to the cell cycle. **C**, **D** The mRNA expression of LARS in LARS knockdown Saos-2 cells (**C**) and LARS knockdown U-2 OS cells (**D**). **E**, **F** The protein expression of LARS in LARS knockdown Saos-2 cells (**E**) and LARS knockdown U-2 OS cells (**F**). **G**, **H** Knockdown LARS inhibited the proliferation of **G** Saos-2 and **H** U-2 OS cells detected by CCK8. **p < 0.01

LARS and DNAJC17 are highly expressed in osteosarcoma. High expression of LARS is an unfavorable prognostic factor in patients with osteosarcoma. However, high expression of DNAJC17 is a favorable prognostic factor in patients with osteosarcoma. Consequently, we were interested in the influence of LARS on osteosarcoma. As noted earlier in this article, a CERES score < − 1 was defined as an essential gene for tumor cell survival. The CERES score of LARS in eight osteosarcoma cell lines was shown in Fig. 7A. The result indicated that the CERES score of LARS was all less than -1 in the above cells. Next, GSEA was performed to identify the differentially activated signaling pathways in the high LARS expression. The GSEA plot showed that high LARS expression positively correlated with the cell cycle (Fig. 7B). These results suggested that LARS may affect the proliferation of osteosarcoma cells. Then, LARS-targeting shRNAs (LARS-shRNA1 and LARS-shRNA2) were transfected into Saos-2 and U-2 OS cells, and LARS was successfully knocked down (Fig. 7C–F). CCK-8 analysis showed that LARS knockdown strongly inhibited the proliferation of Saos-2 and U-2 OS cell lines (Fig. 7G and H).

### Evaluation of associations among LARS and immune cells

**Fig. 8:**
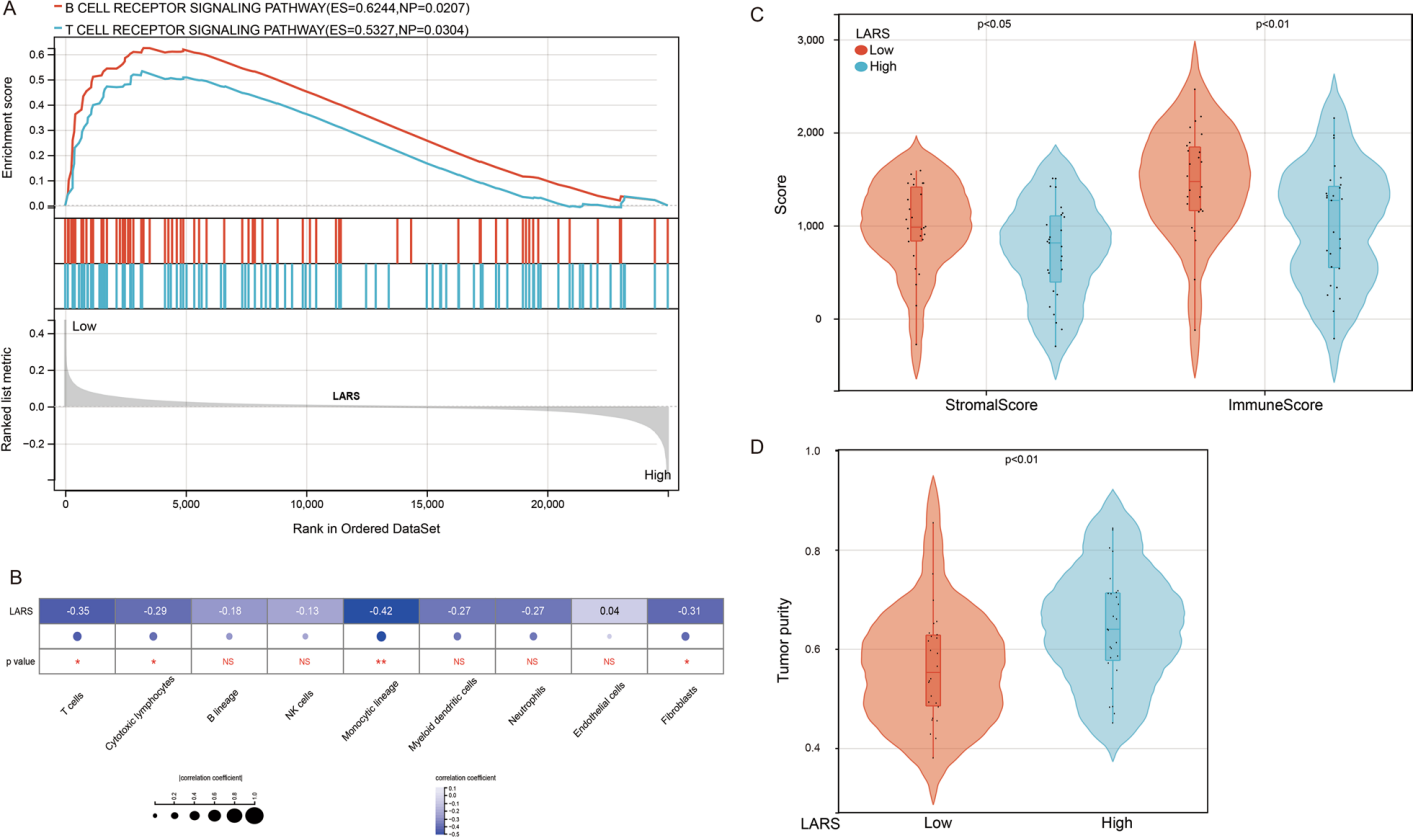
Correlation between LARS expression and the tumor microenvironment in osteosarcoma. **A** GSEA analysis suggested that the high expression of LARS is related to B cell receptor signaling pathway and T cell receptor signaling pathway. **B** Correlation between LARS expression and the number of immune cell infiltrates in osteosarcoma. **C** Correlation between LARS expression and stromal score and immune score in osteosarcoma. **D** Correlation between LARS expression and tumor purity in osteosarcoma. *p < 0.05. **p < 0.01

Our GSEA results showed that high expression of LARS was associated with down-regulation of the B cell receptor signaling pathway and T cell receptor signaling pathway (Fig. 8A). To further validate LARS as a potential immune influencer, we calculated the degree of immune infiltration through MCP-counter to estimate the tendency of residential immune cells to vary with expression changes in LARS. The results indicated that LARS expression is significantly negatively correlated with the infiltration levels of Monocytic lineage (R = − 0.42, p < 0.01), T cells (R = − 0.35, p < 0.05), and Fibroblasts (R = − 0.31, p < 0.05) in osteosarcoma (Fig. 8B). In addition, osteosarcoma patients were classified into low-group and high-group LARS subtypes based on the median LARS expression level. ESTIMATE results indicated that patients with lower LARS expression had a significantly higher stromal score and higher immune score, but with lower tumor purity relative to patients in high-group LARS (Fig. 8C and D).

### The level of LARS protein was highly expressed in osteosarcoma

**Fig. 9:**
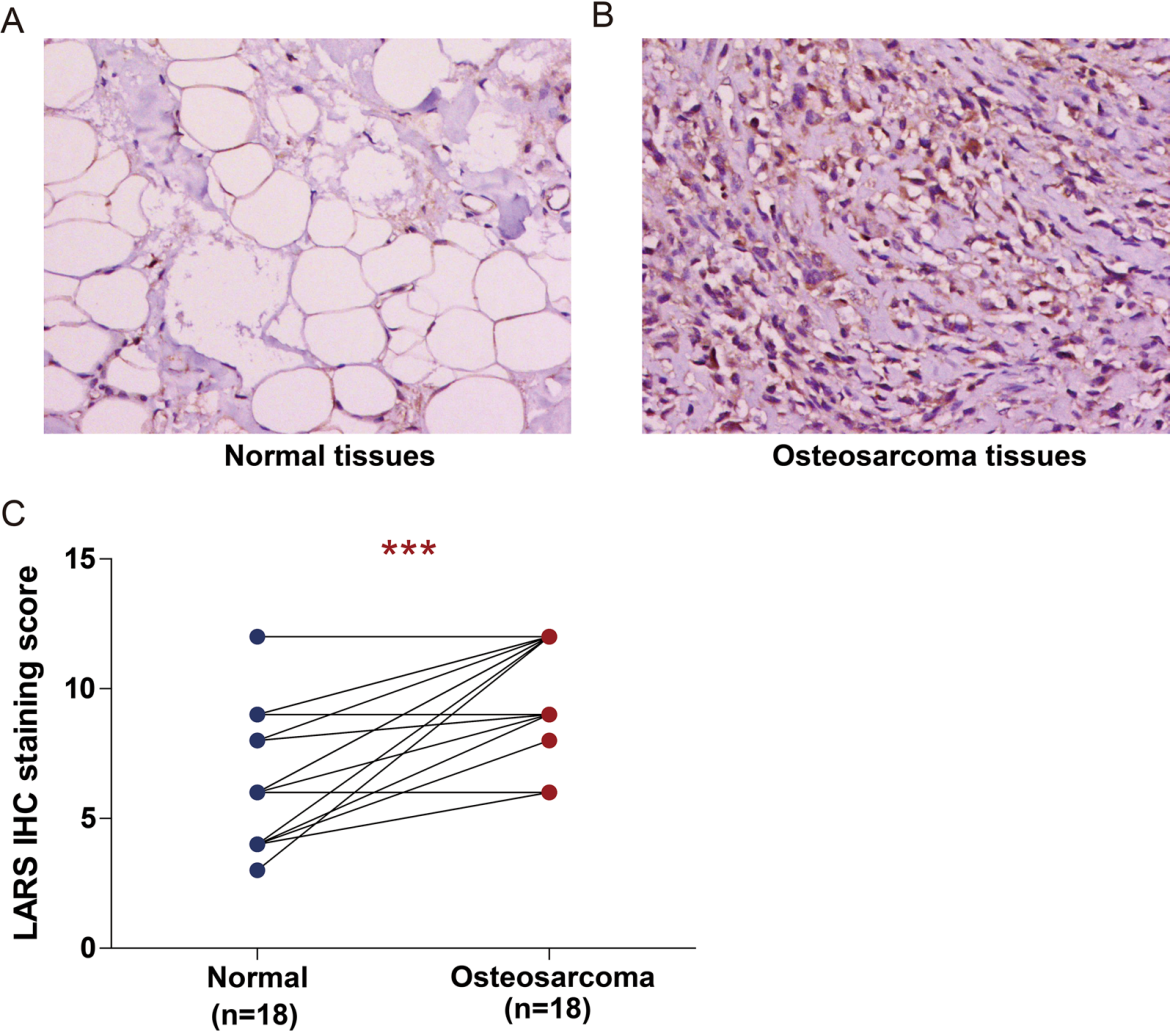
IHC analyses of LARS protein expression in osteosarcoma. **A**, **B** Representative IHC images of LARS protein expression in Normal tissues (**A**) and osteosarcoma tissues (**B**) (×200 magnification). **C** IHC score for LARS in osteosarcoma patients. ***p < 0.001

Finally, the protein expression of LARS in osteosarcoma was detected via IHC staining analysis. LARS protein expression was demonstrated significantly increased in 18 osteosarcoma tissues compared to the paired adjacent normal tissues (Fig. 9A–C).

## Discussion

Osteosarcoma is the most common primary malignant bone tumor, and high morbidity rate and high recurrence rate are the clinical characteristics of osteosarcoma [11]. Osteosarcoma seriously affects the health of adolescents and children [12]. Although great progress has been made in the clinical diagnosis and surgical treatment of osteosarcoma, the overall prognosis of patients with osteosarcoma is still poor. Therefore, it is urgent to find effective molecular therapeutic targets.

Cancer therapeutic target identification has focused on oncogenes and tumor suppressor genes that are mutated in cancer. Mutated oncogenes and tumor suppressor genes that cause cancer also confer properties on cancer cells that differ from normal cells, making them selectively dependent on certain genes for growth or survival. Such genes, which we call selective essential genes, have good specificity as therapeutic targets and are ideal potential therapeutic targets. Therefore, the systematic identification of these selectively essential genes is a novel strategy to identify cancer therapeutic targets. A well-established method for identifying essential genes is to use CRISPER-Cas9 technology to perform genome-wide gene function inactivation screening in cell lines. In recent years, Broad and Anger have performed genome-wide screening of CRISPER-Cas9 in more than 700 cancer cells of different tissue origins and identified essential genes in these cells. Using these data, they constructed the DepMap database, which aims to systematically identify the genetic dependencies of cancer cells.

In the study, the dependence score was calculated by CERES and identified 785 genes essential to the proliferation and survival of osteosarcoma cells from the DepMap website. Of the 785 genes, 59 DEGs were identified in osteosarcoma tissues compared with normal tissues through the GSE19276 database. The results showed that these 59 essential genes were mainly associated with the cell cycle-related signaling pathway in the osteosarcoma tissues. Furthermore, we established a gene signature (LARS and DNAJC17) screened from these 59 genes, and this signature could divide osteosarcoma patients into the low-risk and high-risk groups.

LARS and DNAJC17 are highly expressed in osteosarcoma. High expression of LARS is an unfavorable prognostic factor in patients with osteosarcoma. However, high expression of DNAJC17 is a favorable prognostic factor in patients with osteosarcoma. Consequently, we were interested in the influence of LARS on osteosarcoma.

LARS encodes a cytosolic leucine-tRNA synthetase, a member of the class ARSs (aminoacyl-tRNA synthetase) family. The ARSs family is an extremely ancient enzyme in evolution. Its classic function is to catalyze the esterification reaction between amino acids and their corresponding tRNAs to generate aminoacyl-tRNA, which provides raw materials for protein synthesis in organisms and participates in the production of genetic coding process. The 20 ARSs synthetases correspond to 20 amino acids, and their precise identification of the corresponding tRNA and amino acid substrates ensures the precise transmission of genetic information from mRNA to protein. In the past decades, the identification of cancer-related factors has been an important issue in the field of oncology, not only to understand the basic mechanisms of tumor formation, but also to discover related therapeutic targets. However, ARSs have been neglected, mainly because many people think that ARSs are merely housekeeping genes involved in protein synthesis. Mammalian ARSs have evolved over the course of evolution to develop many additional domains that are not necessarily associated with their catalytic domains. Thanks to these domains, ARSs are able to interact with regulatory factors. ARSs participate in signaling pathways by forming complexes with other regulatory factors [13]. Abnormal expression and mutation of ARSs induce abnormal cellular regulation and protein synthesis. The abnormal function of ARSs is associated with a variety of human diseases, such as autoimmune disorders, metabolic disorders, neuronal diseases, and cancer [14]. Some ARSs are abnormally up-regulated or down-regulated in a variety of tumors [15–24].

Recently, more and more evidences have shown that a variety of ARSs are responsible for non-canonical functions other than enzymatic catalytic activity, such as translation regulation, transcription regulation, synthesis, signal transduction, angiogenesis, inflammation, apoptosis, and the development of cancer [25–29]. The non-classical function of LARS was first reported in 2012. Downregulation of cellular endogenous LARS inhibits mTORC1 activity, thereby affecting cell proliferation [30]. Shin et al. found that the LARS gene is overexpressed in lung cancer cells and tissues, and knockdown of LARS expression can inhibit the growth and metastasis of lung cancer cells. Which suggest that LARS may have carcinogenic potential [31]. However, the expression of LARS was repressed during mammary cell transformation and in human breast cancer. Monoallelic genetic deletion of LARS in mouse mammary glands enhanced breast cancer tumor formation and proliferation. Repression of LARS lead to impaired leucine codon-dependent translation of growth suppressive genes, including gamma-glutamyltransferase 5 (GGT5) and epithelial membrane protein 3 (EMP3). which in turn enhances breast tumor formation and growth [32]. These results indicated that the function of LARS is complex, as it appears to have different functions in different tumors.

In the study, we knocked down LARS gene expression by shRNA. Knockdown of LARS expression inhibited the proliferative ability of osteosarcoma cells, suggesting that LARS has oncogenic potential in osteosarcoma. The correlation between LARS expression and immune cells suggests that LARS regulates osteosarcoma immunity through multiple immune cell populations, such as Monocytic lineage, T cells, and Fibroblasts. The analysis of drug sensitivity revealed that high LARS expression was associated with higher drug sensitivity to BRD-K30748066, Tozasertib, BI-2536, GSK461364, BRD-K70511574, Triazolothiadiazine, Rigosertib, FQI-1, KX2-391 and SB-225002.

Our findings may contribute to further understanding the molecular mechanism and improving the clinical diagnosis and treatment for osteosarcoma. However, limitations of this study still exist including lack of a large cohort of samples from patients with osteosarcoma and short of survival analysis. In addition, further experimental studies are essential for further verifying the mechanisms of LARS in osteosarcoma.

## Conclusion

By analyzing the DepMap database, we identified an essential gene-LARS for survival in osteosarcoma. In addition to its classical function of catalyzing leucyl synthesis, the LARS gene also acts as an oncogene in osteosarcoma cells and is associated with the immune microenvironment of osteosarcoma. LARS may play a key role in the occurrence and progression of osteosarcoma. This study provides new insights into the pathogenesis of osteosarcoma and potential preventive and therapeutic strategies for this condition.

## Supplementary Information

**Additional file 1:** S1. A total of 785 genes critical for maintaining survival in 8 osteosarcoma cell lines.

**Additional file 2:** S2. Fifty-nine genes with significantly altered expression among 785 essential genes in osteosarcoma tissue.

**Additional file 3:** S3. The relationship between the drug sensitivity and the expression of LARS and DNAJC17 based on the data from the GDSC.

## Data Availability

The datasets used and/or analyzed during the current study are available from the corresponding author on reasonable request.
